# Supplementary material for: Is My Cough a Cold or Covid? A Qualitative Study of COVID-19 Symptom Recognition and Attitudes Toward Testing in the UK
Source: Front Public Health. 2021 Aug 18;9:716421. doi: 10.3389/fpubh.2021.716421 (PMC8416344; doi:10.3389/fpubh.2021.716421)
Supplement: Supplementary file 1 [file Data_Sheet_1.PDF]

Interview Topic Guide

Knowledge, perceptions, experience of symptoms

Can you tell me a bit about the last time you had a cold or the flu?

- When was that?
- Were your symptoms mild or severe or somewhere in between?
- How did you manage your symptoms?

Can you tell me about the symptoms of Covid-19? *(see what if any symptoms they cover, only prompt if they don't mention them)*

- Fever: tell me about what you consider to be a fever? How do you measure it?
- Cough: tell me about what you think counts as a cough? How long, how frequent, etc.
- Loss or change to sense of smell/taste: tell me what this means to you? What kind of change?
- Any other symptoms (e.g. sore throat, sneezing, runny nose, headache): What do these mean to you? What would you do about them?

How confident do you feel in identifying symptoms in yourself/your child? If not confident, what might make it easier?

If you have more than 1 child, do you think you would react to symptoms differently depending on which child displayed symptoms?

How confident do you feel being able to tell the difference between Covid-19 and a cold or other illness in yourself/your child? Can you tell me about how you're making that decision?

When would you still go out/send your child to school? What made that important to you?

In what way would how you act on symptoms that you think might be Covid-19 differ from how you would act on symptoms that might not be caused by Covid-19 / have acted on symptoms in the past?

How likely do you feel you/your child are to get Covid-19? Can you tell me about why you do or do not feel at risk?

Have you/your child experienced any of these symptoms (fever, cough, loss/change in taste/smell) in the last 7 days?

- If so, can you tell me step by step about the symptom/s and the experience? Combination of symptoms or just 1? How severe were symptoms?
- What if anything did you do about the symptom(s)? Stay off school/work?
- Did you search for information about the symptom(s)? If no, why not? If yes, what information and where did you look? Was it helpful?

Knowledge, perceptions, experience of testing

Who do you think is eligible to get tested for Covid-19? What makes someone eligible?

When would you ask for a Covid-19 test for yourself/your child?

- How long after symptoms start?
- How many symptoms? Just 1? Combination?

- How severe are the symptoms?

Have you/your child ever had a Covid-19 test?

- If yes:
    - Can you tell me a bit about this experience? What led you to getting a test?
    - Did you have any concerns about getting tested? (data privacy, social stigma or reaction of friends/family/colleagues, missing work/school, loss of income, safety concerns, effectiveness of the test, etc.)?
    - Is there anything that would ever stop you getting a test? Why or why not?
    - Did you/they have any symptoms? If so, what were your/their symptom(s)?
    - When did you seek the test? How long after symptom(s)?
    - How did you get the test? At home, test centre, etc. Could anything make the process easier? If you went to a test centre, did you feel safe?
    - What was the test like? Can you tell me about what you had to do?
    - What happened after you finished the test?
    - How did you get your results?
    - How long were you waiting for your results? Did you do anything differently while you were waiting for your results to come through? How did you feel during this time?
    - Did you feel that people treated you any differently after learning that you'd had a test for coronavirus (regardless of the outcome)?
    - If positive, can you tell me about whether you self-isolated and what this was like? What made it more difficult? What might make it easier? If did not isolate, why not?
    - If positive, did you get in touch with other people to let them know you had tested positive? Did / do they treat you any differently?
    - Were you contacted by Test and Trace? If so, can you tell me a bit about this experience?
- If no:
    - Can you tell me about how you would go about getting a Covid-19 test?
    - Where would you go for information? What information would you want/need?
    - Would you want to be tested? If no, why not? What concerns do you have (data privacy, social stigma or reaction of friends/family/colleagues, missing work/school, loss of income, safety concerns, effectiveness of the test, etc.)?
    - How would you feel about doing the test (would it hurt/be uncomfortable, confident able to do it yourself/for your child)?
    - Would you do anything differently after being tested for coronavirus?
    - Do you think people would treat you differently if you were tested for coronavirus?
        - If positive / If negative
    - Can you tell me about what you would do if the test came back positive?
    - What impact would a positive result have on you/your child's life?

Has anyone you know had a Covid-19 test? What if anything did they tell you about the experience?

What do you think would happen if someone had a positive test result, but did not self-isolate?

What if anything would encourage you to take a test?

What information would you want about testing? Where/who do you want the information coming from? Why? Anyone you do not want information from? Why?

Students only:

Has your university provided any information about testing? If so, can you tell me a bit about this?
